# The Picasso’s skepticism on computer science and the dawn of generative AI: questions after the answers to keep “machines-in-the-loop”

**DOI:** 10.1186/s41747-024-00485-7

**Published:** 2024-07-24

**Authors:** Filippo Pesapane, Renato Cuocolo, Francesco Sardanelli

**Affiliations:** 1 Breast Imaging Division, IEO European Institute of Oncology IRCCS, Milan, Italy; 2 Department of Medicine, Surgery and Dentistry, University of Salerno, Via Salvador Allende 43, Baronissi, 84081 Salerno, Italy; 3 Unit of Radiology, IRCCS Policlinico San Donato, Via Morandi 30, San Donato Milanese, 20097 Milan, Italy; 4 Present Address: Lega Italiana Tumori (LILT) Milano Monza Brianza, Piazzale Gorini 22, 20133 Milan, Italy

## Abstract

Starting from Picasso’s quote (“Computers are useless. They can only give you answers”), we discuss the introduction of generative artificial intelligence (AI), including generative adversarial networks (GANs) and transformer-based architectures such as large language models (LLMs) in radiology, where their potential in reporting, image synthesis, and analysis is notable. However, the need for improvements, evaluations, and regulations prior to clinical use is also clear. Integration of LLMs into clinical workflow needs cautiousness, to avoid or at least mitigate risks associated with false diagnostic suggestions. We highlight challenges in synthetic image generation, inherent biases in AI models, and privacy concerns, stressing the importance of diverse training datasets and robust data privacy measures. We examine the regulatory landscape, including the 2023 Executive Order on AI in the United States and the 2024 AI Act in the European Union, which set standards for AI applications in healthcare. This manuscript contributes to the field by emphasizing the necessity of maintaining the human element in medical procedures while leveraging generative AI, advocating for a “machines-in-the-loop” approach.

More than fifty years ago, the Spanish artist Pablo Picasso made a thought-provoking statement: “Computers are useless. They can only give you answers” [1]. This affirmation implied the idea that computers excel in data processing and efficient calculations, providing useful practice solutions, but they lack the creative intuition and depth of human understanding. However, the landscape has dramatically evolved since then, particularly after the advent of artificial intelligence (AI), and more specifically, generative AI.

Generative AI, which encompasses models like deep learning generative adversarial networks (GANs) and transformers, including large language models (LLMs), has fundamentally shifted the perspective that computers can merely provide answers [2]. In particular, the launch of OpenAI’s ChatGPT (now available as GPT-4) and Google’s Bard (now rebranded to Gemini) in 2022 represented a significant turning point in the accessibility and applicability of LLMs in different scenarios.

The evolution of a computer technology capable of creating high-quality images [3] could have changed Picasso’s notion of computers lacking creativity, now in stark contrast to the capabilities demonstrated by generative AI. Today, we can ask an AI system to create a Picasso-like magnetic resonance image of the brain (Fig. 1), but Picasso would tell us that this is only a “fake Picasso painting” indicating the distinction between AI’s mimicry and genuine human creativity and raising critical questions about the role of AI in fields requiring nuanced judgment and emotional depth, especially in healthcare, where the human element is irreplaceable. Coming to the point, generative AI not only raises philosophical questions about the nature of (human) intelligence and creativity [4], but also practical considerations about the role of machines in human society, including in healthcare, a frontier where AI is making relevant strides. In healthcare, the consequences of incorrect decisions are relevant to the point of saving or not saving human lives, so it is vital to cautiously integrate LLMs.

**Fig. 1:**
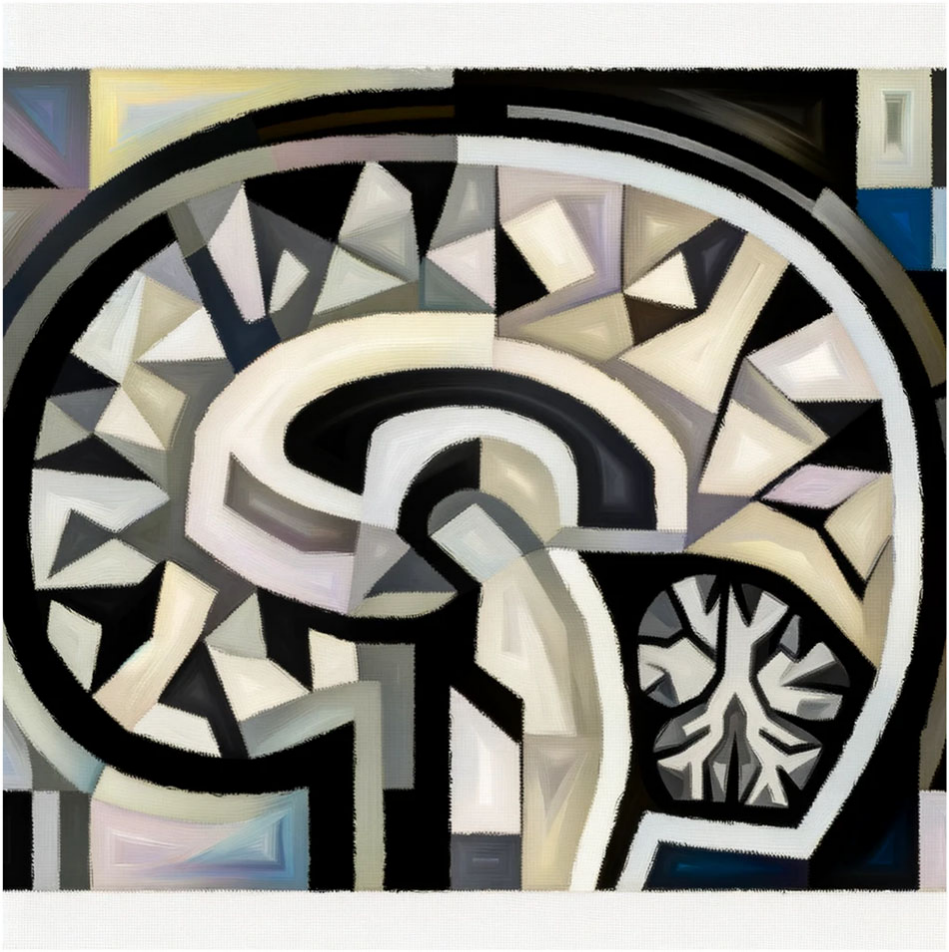
A Picasso-style representation created by OpenAI ChatGPT-4 with the prompt “create a Cubism-like magnetic resonance image of the brain”, showing the potentials of generative AI even in the creation of art pictures

Specialized LLMs, rather than generic ones, are needed in medical settings to avoid the risk of generating unverified diagnostic suggestions. Initiatives like DeepMind’s Pathways Language Model [5] are steps towards creating AI tools attuned to the intricacies of healthcare that are showing promise but still falls short of clinician performance [2], calling for improvements through a collaborative research approach and the adoption of open science practices, including sharing models, code, and publishing results in peer-reviewed journals.

Radiology in particular is witnessing a major growth and development in the last decades, having radiologists always been pioneers of the digital era in medicine and now accepting AI as a new partner in their profession, as predicted in this journal in 2018 [6]. For radiologists, understanding AI is no longer optional but a necessity indeed. This understanding goes beyond mere familiarity. It involves an effort to learn how AI models are developed, trained, and applied in clinical settings. As AI becomes integral to radiological practices, educational programs must evolve to equip professionals with the necessary skills and knowledge, being ready to evaluate the possible role of generative AI integration into their workflow, and assisting radiologists in both clinical and research activities.

An LLM can improve the language quality of a manuscript, making it more readable and clearer, which could be beneficial for non-English native speakers, making knowledge more freely available and producing a better output. The Editors of the three journals of the European Society of Radiology recently published guidelines [7] for the use of LLMs which state the following: 1) AI or AI-assisted technologies cannot be considered as authors; 2) authors must disclose at submission whether they used AI or AI-assisted technologies in their work; 3) authors are fully responsible for any submitted material that includes AI-assisted technologies; 4) any content created by AI or AI-assisted tools must be explicitly labelled; 5) reviewers and editors and should not upload manuscripts to software or other AI-assisted tools where confidentiality cannot be assured. Of note, the Editors say that “generative AI tools will continue to quickly evolve and develop new possibilities in our daily lives”; hence “the statements and policies will need to be re-evaluated and updated regularly” [7].

Unfortunately, as with any technological advancement, generative AI has already shown its potential for the production of spurious scientific papers, with some interesting recent examples. Authors [8] have already shown that LLMs and generative AI can be leveraged to produce completely fake studies, including images, with sufficient quality to pass the peer review process in lower-impact journals. More malevolent actors have already published generative AI-based papers which have, at least in some cases, been retracted [9]. However, the question of how many such papers have not (yet?) been identified remains open. As checklists [10] and quality scoring tools [11] have been made available by scientific journals and societies to address issues in radiomics and AI research, it will be interesting to see how well these apply to generative AI-based manuscripts and published articles and if *ad hoc* tools will become necessary to manage them.

Of note, the application of these AI models extends far beyond linguistic assistance. LLMs look adept in understanding natural language and engaging in multimodal learning, utilizing vast textual and multimodal data to discern connections between medical images, clinical reports, and patient outcomes [12]. The concept of multimodality in AI – processing multiple types of data simultaneously – is particularly promising for radiology: it could significantly enhance the scope of radiological assessments, combining image analysis with patient history, laboratory results, and even genetic data for a holistic approach to diagnosis and treatment planning [2, 12]. In particular, generative AI in medical imaging may overcome limits like limited data diversity and size, costly imaging procedures, and rarity of specific conditions with models that can synthesize diverse images for training and evaluation, enhance image quality through super-resolution or denoising, support processing vast textual and multimodal data and enabling visual-textual data fusion improving clinical decision support [12, 13]. This potential is of particular interest given the growing efforts in sharing medical data across countries, exemplified by the European Health Data Space regulation that is currently being defined for adoption in the near future [14].

On the other hand, generative AI currently presents a significant challenge in the form of hallucinations. Hallucinations occur when the AI produces content that appears plausible but is not grounded in the input data, reflecting its tendency to create responses based on learned patterns rather than factual information. This propensity for generating inaccurate information is particularly concerning in medical applications, where the stakes are high, and errors can lead to serious consequences, including misdiagnoses and inappropriate treatments. The stochastic nature of transformers, which underpin many of these models, means that randomness and hallucinations are inherent aspects of their functioning.

Furthermore, evaluating the performance of LLMs and GANs presents additional challenges due to the lack of suitable performance metrics. Unlike traditional software, where correctness can often be clearly defined and tested, proving the accuracy of generative models is complex and context-dependent. When they are applied to clinical imaging, this complexity is particularly problematic, as the reliability of AI-generated content is critical for clinical use and regulatory approval. Without clear standards and testing protocols, it is difficult to ensure that these models meet the stringent requirements needed for safe and effective healthcare delivery. This ambiguity in performance evaluation, coupled with the absence of robust guidelines, significantly hinders the integration of generative AI into clinical practice, highlighting the urgent need for the development of comprehensive regulatory frameworks and performance benchmarks.

Despite the issue of hallucination, preliminary results have shown that models can support the diagnostic procedure by facilitating tasks such as automated radiology report generation and their interpretation and simplification [15–17], differential diagnosis [18], prediction of disease progression [19], and evaluation of treatment options, extracting patient-centric information from various medical records, finally improving the accuracy and personalization of diagnostic and treatment recommendations [20]. Notably, a study on a gastrointestinal imaging-aware GPT-4 based chatbot [21] has shown that integrating context-specific data into AI models can substantially increase the precision of differential diagnoses for gastrointestinal pathologies, thereby supporting evidence-based clinical decision-making and enhancing the trustworthiness and personalization of healthcare services.

Such advances require that enthusiasm for the potential of generative AI be always accompanied by cautiousness in evaluating their ethical and legal implications. Already in 2018, the spectrum of regulatory and ethical challenges that the integration of AI in radiology “as a medical device” would have been demanded showed non-negligible differences in the European Union (EU) and the United States scenario [22]. Nowadays, the introduction of generative AI into medical research and clinical practice introduces new relevant challenges requiring an update of previous rules to maintain trust in AI-assisted diagnostics.

A critical concern with generative models is the potential for misuse, particularly with images created by GANs: synthetic images could be misused to feign medical conditions or even manipulate clinical trial outcomes, leading to erroneous conclusions and potentially detrimental patient impacts. The high degree of realism in these images could challenge even expert radiologists in distinguishing between authentic and generated images, exacerbating this issue. To mitigate these risks, it is essential to establish guidelines and regulations for the responsible use of AI-generated medical images and to develop techniques for detecting and identifying synthetic images.

Like all the machine/deep learning algorithms, generative models are dependent on extensive datasets for training, which pose risks of inherent biases in the generated images and textual outputs [23]. If the training data lacks representation of the general population or embodies inherent biases, the resultant models could yield biased outcomes, potentially leading to misdiagnoses or inappropriate treatment recommendations [24]. To avert such biases, it is essential to compile diverse and representative training datasets and to meticulously evaluate model performance across various demographic groups [25]. Further, employing bias-mitigation techniques during model training, such as resampling, reweighting, and adversarial training, is crucial to ensure more equitable outcomes [15].

Bigger dataset for training also means higher risk of privacy breaches [24]. When using LLMs tools like OpenAI GPT, patients must be aware that inputting medical information into an online AI program will compromise their privacy protections [26]. Moreover, even in LLMs specialized for healthcare, learned representations might inadvertently capture identifiable patient features, leading to potential leaks of personal information [13, 24]. Robust data privacy measures, data anonymization, and de-identification are crucial to prevent breaches. Such requirements were already introduced in the past years with regulations such as the “Health Insurance Portability and Accountability Act” in the United States and the “General Data Protection Regulation” in the EU [22]. Important to note, one main limitation of GDPR is that this regulation has been thought to personal data in “general” (as announced by the first word of its denomination), not specifically for medical data [22, 27]. This oversight challenges the balance between protecting individual privacy rights and fostering innovation in healthcare through data utilization.

New regulations have been approved in the light of generative AI’s advent. In EU, the recently approved “Artificial Intelligence Act” [28] introduces specific regulations for critical sectors, like healthcare, to assess the impact of AI on fundamental rights, including conducting mandatory assessments for high-risk AI systems. These systems, potentially encompassing advanced medical imaging AI, must undergo rigorous evaluations to mitigate systemic risks and ensure cybersecurity. This “AI Act” defines AI systems as software capable of generating outputs that influence their interaction environments: consequently, medical device software, including diagnostic aids and personalized treatment recommendations, falls under this definition, necessitating compliance with the AI Act. Devices classified as high-risk must meet specific conditions, such as being a safety component of a product covered by EU harmonization legislation, which includes the “Medical Device Regulation” [29] and the “In Vitro Diagnostic Devices Regulation” [30]. Software intended for diagnostic or therapeutic decision-making will be classified accordingly, with most falling into higher risk categories, thereby subjecting them to the AI Act’s stringent requirements. A risk-based approach permits high-risk AI systems in the European market, provided they fulfill mandatory requirements and undergo an *ex ante* conformity assessment. This assessment integrates with existing sectoral legislation procedures, such as those for medical devices, to avoid duplicative efforts and streamline compliance.

The intertwining of the AI Act with personal data protection laws, notably the GDPR, is inevitable given the extensive data processing AI systems entail. This raises complexities in data processing legality, especially concerning data accuracy and the quality of input data—crucial for AI’s effectiveness. The AI Act stipulates that AI systems must be developed with high-quality training, validation, and testing datasets, potentially necessitating personal data processing [28]. This processing, particularly of sensitive categories under GDPR, must adhere to stringent safeguards, highlighting challenges in data transfer and jurisdictional conflicts.

The incorporation of AI Act assessments into existing medical device conformity assessments aims to minimize bureaucracy. However, at the moment challenges still remain, such as the capacity of notified bodies to evaluate AI systems, potentially leading to increased certification timelines and costs [31].

In the United States, the 2023 Executive Order on AI [32] set standards for AI, addressing data protection and discrimination for safe and trustworthy AI systems, seeking to promote innovation while ensuring responsible government use of AI technologies. The order includes measures for protecting personal data, especially of minors, and addresses the potential for AI to exacerbate discrimination and bias. The Executive Order specifically mandates the Department of Health & Human Services to undertake several critical initiatives covering a broad spectrum of applications, including diagnostics, drug and medical device development, personalized care delivery, and patient monitoring. These include the formation of an AI Task Force aimed at developing a strategic framework for the ethical deployment and use of AI across health and human services sectors. The Task Force’s areas of focus span research and discovery, drug and device safety, healthcare delivery and financing, and public health. Furthermore, the Department of Health & Human Services is tasked with creating a quality assurance policy for AI in healthcare, advancing compliance with federal nondiscrimination laws, establishing an AI safety program, and developing regulatory strategies for AI in drug development, including thorough premarket assessments and continuous post-market oversight.

On November 3, 2023, the Office of Management and Budget responded to the Executive Order by releasing a draft policy for public comment [33]. This draft outline new requirements for AI governance, innovation, and risk management, emphasizing the need for specific risk management practices for AI applications that affect public safety. These initiatives indicate a proactive approach by the government to manage the complex interplay between AI innovation and the regulatory frameworks necessary to ensure these technologies are developed and implemented responsibly in the healthcare sector. Stakeholders in the healthcare and technology industries should anticipate forthcoming guidance from Health and Human Services, the Food and Drug Administration, and other relevant agencies, reflecting an ambitious timeline for implementing these critical directives.

For radiologists, regulations mean an increased emphasis on ensuring that AI applications are safe, transparent, nondiscriminatory, and respect fundamental rights, ensuring that generative AI in medical imaging transcends mere technological advancement, ensuring it is ethically sound and legally compliant.

Finally, as radiologists will start to integrate generative AI into their practice, it is crucial they follow best practices, including data privacy, model validation, ethical AI use, and open science principles, always preserving their humanistic approach in their work. The words attributed to the American journalist S.J. Harris sound particularly pertinent: “The real danger is not that computers will begin to think like men, but that men will begin to think like computers” [34]. While generative AI offers the ability to mimic the extensive knowledge (and perhaps also empathy?) of a seasoned physician, providing timely answers and facilitating doctor-patient communication through the integration of medical images and radiology reports [16, 17], radiologists must exercise vigilance. They be mindful not to delegate to technology, regardless of its effectiveness, the core of their profession: the assistance to the patient as a person and not as a disease, the need to cure the patient and not her/his images [35]. Their focus should not be on using this technology as a means to save time or effort, but to use such tools to provide better assistance to the patient, ensuring that the essential human element in medicine is preserved.

We should always remember that the advent of generative AI did not change the basic scenario: AI systems do not understand what they do. They (still?) lack common sense as well as the ability to elaborate abstractions and reasoning by subtle analogies, capacities typical of human natural intelligence, as already explained by M. Mitchell in 2019 [36].

In conclusion, while Picasso’s statement on the computers as mere answer-giving machines was reflective of his time, the advent of generative AI is changing this perspective, introducing in a new era where computers are not only tools for computation but also partners in more intellectual and creativity activities. Generative AI is such a recent innovation that radiologists—like the other healthcare providers—are all still learning. It has only been out in the world since late 2022, and medicine usually takes many years to put new discoveries to widespread use. The current role of AI in radiology is the tip of the iceberg, with the integration of generative AI marking the start of a vast and promising future in clinical application. With rigorous scientific validation and ethical oversight, the incorporation of AI into radiology promises to be a pivotal shift towards a more efficient, accurate, and patient-centric approach, ensuring that technology enhances the most valuable aspects of the radiological profession.

Even though AI seems to be increasingly able to give appropriate answers, the role of questions coming from humans remains crucial. The Norwegian writer and philosopher Joestein Gaarder wrote [37]: “An answer is always the stretch of road that’s behind you. Only a question can point the way forward”. Our ability to ask question (to ourselves other than to machines again) will allow us to close the circle, keeping “machines-in-the-loop”, not to be kept as “humans-in-the loop” by machines.
